# Photoreceptors inhibit pathological retinal angiogenesis through transcriptional regulation of Adam17 via c-Fos

**DOI:** 10.1007/s10456-024-09912-0

**Published:** 2024-03-14

**Authors:** Xudong Wang, Tianxi Wang, Satoshi Kaneko, Emil Kriukov, Enton Lam, Manon Szczepan, Jasmine Chen, Austin Gregg, Xingyan Wang, Angeles Fernandez-Gonzalez, S. Alex Mitsialis, Stella Kourembanas, Petr Baranov, Ye Sun

**Affiliations:** 1 Department of Ophthalmology, Boston Children’s Hospital, Harvard Medical School, Boston, MA USA; 2 Department of Ophthalmology, The Schepens Eye Research Institute of Massachusetts Eye and Ear, Harvard Medical School, Boston, MA USA; 3 Division of Newborn Medicine, Department of Pediatrics, Boston Children’s Hospital, Harvard Medical School, Boston, MA USA

**Keywords:** Photoreceptor, Retinal angiogenesis, c-Fos, Adam17

## Abstract

Pathological retinal angiogenesis profoundly impacts visual function in vascular eye diseases, such as retinopathy of prematurity (ROP) in preterm infants and age-related macular degeneration in the elderly. While the involvement of photoreceptors in these diseases is recognized, the underlying mechanisms remain unclear. This study delved into the pivotal role of photoreceptors in regulating abnormal retinal blood vessel growth using an oxygen-induced retinopathy (OIR) mouse model through the c-Fos/A disintegrin and metalloprotease 17 (Adam17) axis. Our findings revealed a significant induction of c-Fos expression in rod photoreceptors, and c-Fos depletion in these cells inhibited pathological neovascularization and reduced blood vessel leakage in the OIR mouse model. Mechanistically, c-Fos directly regulated the transcription of *Adam17* a shedding protease responsible for the production of bioactive molecules involved in inflammation, angiogenesis, and cell adhesion and migration. Furthermore, we demonstrated the therapeutic potential by using an adeno-associated virus carrying a rod photoreceptor-specific short hairpin RNA against *c-fos* which effectively mitigated abnormal retinal blood vessel overgrowth, restored retinal thickness, and improved electroretinographic (ERG) responses. In conclusion, this study highlights the significance of photoreceptor c-Fos in ROP pathology, offering a novel perspective for the treatment of this disease.

**Supplementary Information:**

The online version contains supplementary material available at 10.1007/s10456-024-09912-0.

## Introduction

Retinal photoreceptors, specialized neurons in the eyes, are pivotal components of our visual system, responsible for translating changing light patterns into electrical signals [1]. To safeguard these crucial elements, mammals have developed a unique defense mechanism known as immune privilege, which shields the ocular system from the systemic circulation. This protection ensures that retinal neurons remain unharmed by pathogenic infections or damages, preserving the integrity of the retina and our precious sense of light. However, in numerous vascular ocular diseases that frequently afflict individuals, this immune privilege can be compromised. When this occurs, it can lead to chronic inflammation and abnormal angiogenesis within the eye, ultimately resulting in retinopathy and vison loss. Recent findings have shed light on an intriguing revelation: photoreceptors emerge as a significant source of inflammatory factors under specific health conditions. For instance, in diabetic retinopathy mouse models [2–4] and very low-density lipoprotein receptor knockout (*Vldlr*^*−/−*^) mice [5], a model used to study pathological angiogenesis invading the photoreceptor avascular zone [6–10], photoreceptors have been found to actively participate in the immune response. This underscores the importance of immune regulation originating from photoreceptors in the pathology of vascular ocular diseases. Despite these exciting discoveries, the underlying molecular mechanisms are poorly understood. We are only at the initial stages of comprehending how photoreceptors contribute to immune responses in the context of these diseases. This knowledge gap underscores the need for further exploration and research to fully elucidate these mechanisms and, ultimately, unlock the potential for innovative treatments that could transform the management of vascular ocular diseases.

To uncover the intricate molecular mechanisms governing immune regulation originating from photoreceptors, a suitable mouse model is essential. The oxygen-induced retinopathy (OIR) [11–14] is a widely used mouse model for retinopathy of prematurity (ROP), a primary cause of childhood blindness in preterm infants characterized by abnormal overgrowth of retinal blood vessels. ROP progression begins with vascular attenuation due to hyperoxia after birth (Phase I), followed by excessive production of vascular growth factors and neovascularization (NV) (Phase II) as the retina develops in relative hypoxia [15]. In ROP patients, NV typically begins around 32-week gestational age [16, 17], regardless of the gestational age at birth, coinciding with the maturation of rod photoreceptor outer segments and full rod photoreceptor function [18]. ROP patients exhibit varying electroretinography (ERG) responses to the severity of acute phase of ROP, indicating the involvement of rod photoreceptors ROP [19]. Studies in *Pdeb*^*rd1*^ mutant mice with photoreceptor cell degeneration have shown that they do not develop NV in the OIR model [20]. These findings highlight the OIR model as a valuable tool for investigating the molecular mechanisms underlying immune regulation originating from photoreceptors in the pathology of vascular ocular diseases.

The transcription factor c-Fos encodes a nuclear phosphoprotein that forms heterodimeric complexes with Jun-family molecules to form the activator protein 1 complex (AP-1 complex) [21, 22]. This intricate complex functions as a master regulator, orchestrating numerous inflammatory signaling pathways [23], akin to its involvement in diseases like rheumatoid arthritis [24, 25]. c-Fos is expressed rapidly and transiently across various cell types in response to a wide range of external stimuli [26]. Notably, c-Fos has been identified in the retinal photoreceptors of 26–40 week old human fetuses [27]. Under specific conditions, such as darkness, the expression *c-fos* is induced in photoreceptors [22]. This highlights the adaptability of c-Fos, which can be activated under various circumstances. In our study, utilizing the OIR mouse model, we made a significant discovery in the context of retina angiogenesis. We observed a substantial upregulation of *c-fos* gene expression in the rod photoreceptor cells in the OIR mouse model. Notably, when we selectively deleted *c-fos* in rod photoreceptors within the OIR mouse model, we observed a remarkable reduction in pathological neovascular tufts and decreased blood vessel leakage. This provided compelling evidence of the direct involvement of photoreceptor c-Fos in the regulation of retinal blood vessel growth. Delving deeper into the molecular underpinnings, our investigations involved techniques including CUT&Tag sequencing analysis and luciferase reporter assays. Through these methods, we identified that c-Fos directly regulated the expression of A disintegrin and metalloprotease 17 (*Adam17*), a protease responsible for shedding activities. Furthermore, to investigate the therapeutic potential of targeting c-Fos, we employed subretinal injection of adeno-associated virus (AAV) containing *c-fos* shRNA, specifically targeting rod photoreceptor cells. This intervention yielded remarkable results by attenuating OIR pathology, restoring retinal thickness, and improving ERG responses. In summary, we discovered a central role of photoreceptor c-Fos/Adam17 in the complex processes of retinal angiogenesis, providing a novel perspective for the treatment of vascular eye diseases.

## Materials and methods

### Animals

All mouse studies were reviewed and approved by the Institutional Animal Care and Use Committee (IACUC) at the Boston Children’s Hospital and all mouse experiments were performed following the guidance of The Association for Research in Vision and Ophthalmology (ARVO) for the ethical use of animals in ophthalmic and vision research. *c-fos* floxed (*c-fos*^*f/f*^) mice (Jackson Laboratory, stock #037115-JAX) was crossed with rod photoreceptor-specific rhodopsin Cre mice (*Rho*^*iCre*^) (Jackson Laboratory, stock #015850) and rod photoreceptor-specific Nrl Cre mice (*Nrl*^*Cre*^)(Jackson Laboratory, stock #028941), respectively to generate rod photoreceptor-specific *c-fos* knockout mice. Both male and female mice were used for all experiments. C57BL/6J mice (Jackson Laboratory, stock # 000664) were obtained from the Jackson Laboratory.

### Oxygen-induced retinopathy (OIR) and NV quantification

OIR was induced in neonatal mice as described previously [11, 28]. In brief, mouse pups were placed with nursing female mice and exposed to 75% oxygen from postnatal day (P) 7 to P12. Subsequently, they were returned to room air until P17 for phenotypical analysis and gene expression at the designated time points. For phenotypical analysis, eyes were collected at P17, dissected, and stained with fluorescent dye conjugated- Griffonia Simplicifolia Isolectin IB4 (Invitrogen) overnight, and flat-mounted. The avascular (VO) and pathological NV areas were quantified [13] by researchers who were blinded to the conditions, using Image J (National Institutes of Health, http://imagej.nih.gov/ij/) and Adobe Photoshop (Adobe Systems). The exclusion criterion for OIR mouse model was used as described previously, wherein mice with a body weight of less than 5 g at P17 were excluded from the study [29].

### Preparation of AAV2-CAG*-shc-fos* vector and AAV

Four independent shRNAs against mouse *c-fos* were designed using a published algorithm [30]. The sequences of the mouse *c-fos* shRNAs are described previously [5]. The shRNAs were cloned into a CAGmiR30-GFP plasmid to assess their knockdown efficiency in pup retinas and photoreceptor 661 W cells. Recombinant AAV2 vectors were produced as previously described [31, 32]. In brief, AAV vector, rep/cap packaging plasmid, and adenoviral helper plasmid were combined with polyethylenimine (Sigma) and transfected into HEK293T cells (catalog HCL4517; Thermo Scientific). After 72 h of transfection, cells were harvested, and the cell pellet was resuspended in virus buffer. Subsequently, the suspension underwent three cycles of freeze-thaw and homogenization using a Dounce homogenizer. Cellular debris was pelleted at 5,000 g for 20 min, and the supernatant was layered on an iodixanol gradient. Recovered AAV vectors were washed 3 times with PBS using Amicon 100 K columns (EMD Millipore). Real-time PCR was employed to determine genome titers of the recombinant AAV. This protocol was also used to prepare a control AAV2-shControl. The viruses were diluted to various concentrations for infection tests, with a concentration of approximately 2 × 10^12^ gc/ml used for the experiments.

### Subretinal injection

Subretinal injection into the eyes of neonatal P1 mice was performed as previously described [33, 34] under a dissection microscope. P1 pups were briefly anesthetized on ice. The eyelid was prepared by applying Betadine, followed by water and then 70% ethanol using cotton swabs. A blade was used to gently create an incision in the eyelid. The injection needle was carefully inserted into the eyeball through the incision until slight resistance was encountered. Approximately 0.5 µl solution containing AAV (at a concentration of 10^12^–10^13^ gc/ml) was introduced into the subretinal space using a pulled angled glass pipette controlled by a FemtoJet (Eppendorf). Curved forceps were employed to slowly close the eyelid. The mice were then placed on a circulating water blanket to maintain warmth. Retinas were collected at P17 for whole-mount analysis.

### Fundus fluorescein angiography (FFA)

FFA was performed as described previously [5]. In brief, mice were anesthetized and injected intraperitoneally with fluorescein AK-FLUOR (Akorn) at 5 µg/g body weight. Fluorescent fundus images with dilated pupils were taken with a retinal-imaging microscope (Micron IV; Phoenix Research Laboratories) at 2 and 8 min after fluorescein injection.

### Electroretinography (ERG)

Retinal function was assessed by ERG as previously described [35]. In brief, dark-adapted, anesthetized (ketamine/xylazine) mouse pupils were dilated (Cyclomydril; Alcon, Fort Worth, TX), and their corneas were anesthetized (proparacaine). A Burian–Allen bipolar electrode designed for the mouse eye (Hansen Laboratories, Coralville, IA) was placed on the cornea, and the ground electrode was placed on a mouse foot. The stimuli consisted of a series of “green” LED flashes of doubling intensity from ~ 0.0064 to ~ 2.05 cd⋅s⋅m-2 and then “white” xenon-arc flashes from ~ 8.2 to ~ 1050 cd⋅s⋅m-2. The “equivalent light” for the green and white stimuli was determined from the shift of the stimulus/response curves. ERG stimuli were delivered using a Colordome Ganzfeld stimulator (Diagnosys LLC, Lowell, MA). The saturating sensitivity of the rod photo response was estimated by fitting a model of the biochemical processes involved in the activation of phototransduction to the ERG a-waves [36–38].

### In vivo imaging using optical coherence tomography (OCT)

Mice were anesthetized with a mixture of xylazine (6 mg/kg) and ketamine (100 mg/kg), and pupils were dilated with a topical drop of Cyclomydril (Alcon Laboratories). Two minutes after pupil dilation, lubricating eye drops (Alcon Laboratories) were applied to the cornea. Spectral domain optical coherence tomography (OCT) with guidance of bright-field live fundus image was performed using the image-guided OCT system (Micron IV, Phoenix Research Laboratories) according to the manufacturer’s instruction and using the vendor’s image acquisition software to generate fundus images and OCT scans. The vendor’s software Insight was used to accurately measure the thickness of retinal layers and entire retinas. The thickness of retinal layers was plotted with 4 distances from the optic nerve head (100, 200, 300, 400 μm).

### RNA isolation and quantitative RT-PCR

Total RNA was extracted from mouse retina using Quick-RNA™ Miniprep Kit (Zymo Research, R1054). cDNA was synthesized using iScript™ cDNA Synthesis Kit (Bio-Rad, 1708890). Quantitative PCR (qPCR) was performed using SYBR Green qPCR Master Mix (Apex Bio, K1070). Primer sequences used were: *c-fos*, forward 5’-GGG GAC AGC CTT TCC TAC TA-3’ and reverse 5’-CTG TCA CCG TGG GGA TAA AG-3’; *Il6*, forward 5’-TAG TCC TTC CTA CCC CAA TTT CC-3’ and reverse 5’-TAG TCC TTC CTA CCC CAA TTT CC-3’; *Stat3*, forward 5’-GGC ACC TTG GAT TGA GAG TC-3’ and reverse 5’-CGA AGG TTG TGC TGA TAG AGG-3’; *Tnf*, forward 5’-CGA AGG TTG TGC TGA TAG AGG-3’ and reverse 5’-TGG GAG TAG ACA AGG TAC AAC CC-3’; *Vegfa*, forward 5’-TGG GAG TAG ACA AGG TAC AAC CC-3’ and reverse 5’-GGC GAT TTA GCA GCA GAT ATA AGA A-3’.

### Immunohistochemistry

Immunostaining in retinas was performed as described [14]. In brief, eyes were isolated from P17 OIR mice, fixed and permeabilized. The flat-mounted retinas or cross sections were stained with antibodies and imaged using a confocal laser scanning microscope (Olympus FV1000 and Zeiss LSM980).

### Immunoblot

A standard immunoblotting protocol was used. In brief, a buffer containing 300 mM NaCl, 0.5% NP-40, 50 mM Tris-HCl pH 7.4, 0.5 mM EDTA was used to lyse the retinas. Proteinase and phosphatase inhibitor cocktails were added. The antibodies included c-Fos (9F6) Rabbit mAb (Cell Signaling Technology 2250 S, RRID# AB_2247211), Adam17 antibody (Abcam ab13535, RRID# AB_300436), and β-Actin (D6A8) Rabbit mAb (Cell Signaling Technology, 8457 S, RRID# AB_10950489).

### Luciferase reporter constructs preparation and luciferase reporter assays

DNA fragments containing putative AP-1 binding site in *Adam17* promoter regions were amplified from mouse genomic DNA with primers (forward 5’-ATA GCT AGC GTG GCG CAC GCC TTT AAT CC-3’ and reverse 5’-GAG AGA TCT CAC GTC CCC GGA AGT GC-3’), then cloned into luciferase report vector *pTA-luc* (Clontech). Mutant constructs of *Adam17* with deletion in AP-1 sites were also constructed with luciferase reporter. HEK293T cells were transiently transfected with c-Fos expressing plasmid (pcDNA3-FLAG-FosWT, Addgene # 8966) and c-Jun expressing plasmid (Flag-JunWT-Myc, Addgene: 47,443), wild type or mutant *pTA-luc-Adam17* promoter reporter, or Renilla luciferase vector (pRL-SV40, Promega) alone as control. At 36 h post-transfection, cells were lysed in passive lysis buffer (Promega, Dual-Luciferase assay system) and luciferase reporter activities were measured using a EnSight multimode plate reader (Perkin Elmer).

### CUT&Tag sequencing and analysis

CUT&Tag was performed as described [39]. In brief, freshly isolated retinas from three mice were minced and pooled as a group sample. The pooled retinas were incubated in NE1 buffer (20 mM HEPES pH7.8, 10 mM KCl, 0.5 mM spermidine, 0.1% Triton X-100, 20% glycerol, Roche proteinase inhibitor) for 10 min on ice, then homogenized by Dounce homogenizer. The suspension was centrifuged at 100 x g for 30 s at 4 °C. The supernatant containing nuclei was transferred to a fresh tube. The nuclei number was counted by a hemacytometer. Half a million nuclei were conjugated to activated concanavalin A-coated magnetic beads (Bangs Laboratories, BP531). The beads were separated by a magnet stand and resuspended in Wash buffer (20 mM HEPES pH 7.5, 150 mM NaCl, 2.0 mM EDTA, 0.5 mM spermidine, 0.01% digitonin, Roche proteinase inhibitor) with primary antibody (1:50 dilution). After incubation at 4 °C overnight on a rotator, the beads were separated, resuspended in Wash buffer with secondary antibody (1:100 dilution), and incubated for 30 min at RT on a rotator. The antibodies used CUT&Tag were listed in the supplementary Table S1. The beads were washed with Wash buffer once and resuspended in Wash buffer (20 mM HEPES pH 7.5 300 mM NaCl, 0.5 mM spermidine, 0.01% digitonin, Roche proteinase inhibitor) with pAG-Tn5 (EpiCypher, 15-1017). After incubation for 1 h at RT, the beads were washed with Wash buffer while the beads were on a magnet stand. For tagmentation of DNA, the beads were resuspended in Tagmentation buffer (Wash buffer with 10 mM MgCl2) and incubated for 1 h at 37 °C. The tagmentation was eliminated by adding 0.5 M EDTA, 10% SDS, and 20 µg/µl proteinase K (final concentration 16 mM EDTA, 0.1% SDS, 0.16 µg/µl proteinase K) with incubation for 10 min at 70 °C. The tagmented DNA was extracted by phenol-chloroform extraction, precipitated by ethanol precipitation, and dissolved in 21 µl TE buffer containing 1 mM Tris pH 8.0, 0.1 mM EDTA. To prepare CUT&Tag libraries, 2 µl of 10 µM i5 primer, 2 µl of 10 µM barcoded i7 primer, and 25 µl of NEBNext HiFi 2x Master mix (NEB, M0541L) were added. PCR was performed for 12 cycles. Following the manufacturer’s instructions, the libraries were purified using AMPure XP bead (Beckman Coulter, A63880). The concentration of libraries was measured by KAPA library quantification kit (Roche, KK4844) following the manufacturer’s instructions. Paired-end 150 reads sequencing of the pooled libraries was used by the Illumina NextSeq550 system high-output kit. Data processing and analysis were performed using an automated chromatin Immunoprecipitation sequencing analysis pipeline, ChIP-AP (https://github.com/JSuryatenggara/ChIP-AP) [40]. For visualization of CUT&Tag tracks, the integrative genomics viewer (IGV) was used. Excel and JMP Pro were used for the Venn diagram.

### scRNA-seq analysis

OIR model mice scRNA-sec dataset (GSE150703) was obtained from the Gene Expression Omnibus (GEO). Retinal cells were classified by hierarchical clustering (JMP Pro) with retinal cell makers, t-SNE clustering (R package Rtsne), and UMAP clustering (R package umap). The rod photoreceptor cell’s scRNA-seq data were separated and utilized for further analysis. Differentially expressed genes (DEGs) and Venn diagram were analyzed in Excel and visualized with JMP Pro.

### Statistical analysis

GraphPad Prism was used for statistical analysis. Results are presented as means ± Standard Error of the Mean (SEM). All experiments were repeated independently at least three times. Mann-Whitney test was used for two-group comparison. Kruskal-Wallis Dunn’s test was used for multiple-group comparison. *P* values < 0.05 were considered statistically significant.

## Results

### c-Fos was both induced and activated in the photoreceptor layer during NV development

**Fig. 1:**
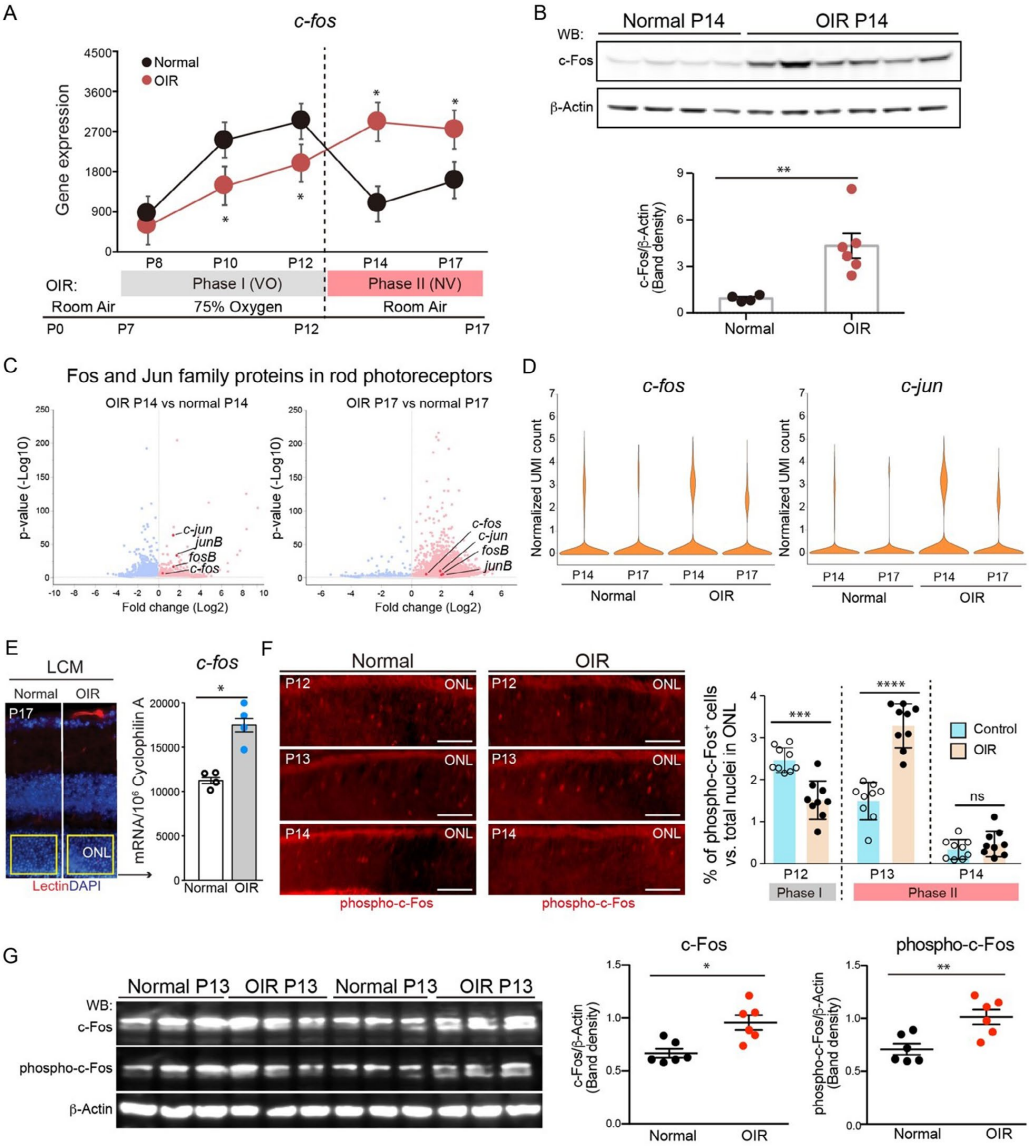
c-Fos was induced and activated in rods during OIR. (**A**) *c-fos* mRNA levels were assessed in whole retinas from both normal and OIR mice using RT-qPCR (*n* = 6). (**B**) Western blots were conducted to examine c-Fos protein expression in P14 retinas from both normal and OIR mice (*n* = 4–6). The levels of c-Fos were determined by quantifying the density of c-Fos bands and normalized them to β-Actin. (**C**) Differentially expressed genes (DEGs) in rod photoreceptor cells from OIR and normal mice were analyzed using publicly available scRNA-seq dataset (GSE150703). AP-1 family genes, including *c-fos, fosB, c-jun*, and *junB*, were highlighted and indicated. (**D**) The expression patterns of *c-fos* and *c-jun* in rod photoreceptor cells under OIR and normal conditions were examined using the scRNA-seq dataset (GSE150703). (**E**) Laser-induced micro-sectioning of the outer nuclear layer (ONL) was performed, followed by qPCR analysis of *c-fos* expression (*n* = 4). (**F**) Immunofluorescence staining was employed to visualize c-Fos phosphorylation in the ONL of normal and OIR retinas. Quantification of phosphor-c-Fos in the rod photoreceptor cells was conducted. The % of phosphor-c-Fos^+^ cells was calculated based on three fields of a retina section (*n* = 10–12). (**G**) Western blots were conducted to examine phosphorylation of c-Fos (phospho-c-Fos) and total c-Fos expression in P14 retinas from both normal and OIR mice (*n* = 6). Data are represented as mean ± SEM. Mann-Whitney test was used for two-group comparison. Kruskal-Wallis Dunn’s test was used for multiple-group comparison. ***, p < 0.001; **, *p* < 0.01; *, *p* < 0.05

To explore the potential role of c-Fos in the context of retinopathy, we initiated our investigation by assessing the mRNA expression of the *c-fos* gene in retinal tissues, both under normal condition and in the presence of OIR. In the OIR mouse model, a two-phase exposure was employed: firstly, mice experienced a hyperoxia stage by being subjected to 75% oxygen levels from postnatal day (P) 7 to P12, known as Phase I vaso-obliteration (VO). Subsequently, they were transitioned to room air with 21% oxygen for an additional 5 days from P12 to P17, representing the hypoxia stage, or Phase II NV. Comparative analysis revealed a noteworthy reduction in *c-fos* expression during the VO stage, followed by a significant increase during the NV stage in OIR retinas when compared to normal controls (Fig. 1A). Furthermore, c-Fos protein level increased at P14 in OIR retinas, as validated by western blot analysis (Fig. 1B), suggesting its critical role in the OIR process.

We next analyzed the expression of Fos family proteins within rod photoreceptors by leveraging a single-cell RNA sequencing (scRNA-seq) dataset (GSE150703). Our analysis revealed elevated levels of *c-fos, fosB, c-jun*, and *junB* during the NV stage at P14 and P17 (Fig. 1C, D). To further validate the specific induction of *c-fos* in rod photoreceptors during NV development, we utilized laser-captured microdissection as described [14] to isolate the photoreceptor layer. The examination of *c-fos* expression in photoreceptor cells during OIR revealed a significant increase in mRNA level in the outer nuclear layer (ONL) of OIR retinas compared to normal retinas during NV development (Fig. 1E).

Phosphorylation of c-Fos by extracellular signal-regulated protein kinase (ERK) can enhance its transcriptional activity [41]. To assess c-Fos activation during NV, we quantified the level of phosphorylated c-Fos using immunostaining with phospho-c-Fos antibody. In normal retinas, phosphorylated c-Fos was detected in the ONL at P12 and gradually decreased at P13 and P14, suggesting a reduced requirement for c-Fos after the completion of retinal photoreceptor development around P12 [42, 43]. In contrast, OIR retinas exhibited a distinct pattern: the numbers of phosphorylated c-Fos positive cells were significantly reduced at P12 during hyperoxia (Phase I) compared to normal retinas but rapidly increased at P13 when the mice were exposure to hypoxia, indicating a critical role of c-Fos during hypoxic condition (Fig. 1F). Increased phospho-c-Fos at P13 OIR retinas compared to normal retinas was further confirmed with western blots (Fig. 1G). Collectively, our data underscored the dynamic changes in c-Fos expression and activation in rod photoreceptors during OIR, implicating its significance in NV development.

### Photoreceptor specific c-Fos deletion effectively suppressed retinal NV and mitigated blood vessel leakage in the OIR model

**Fig. 2:**
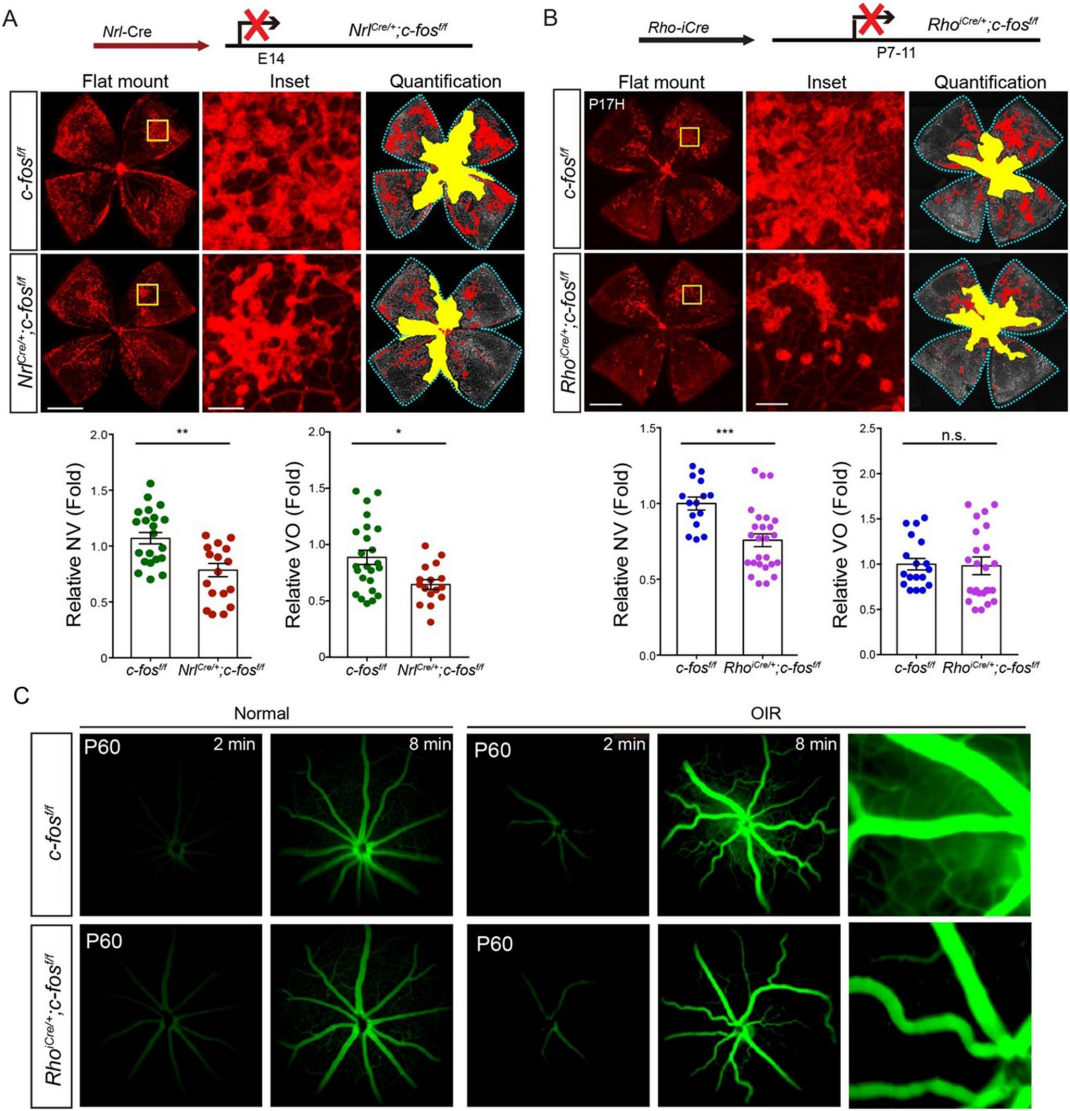
Rod c-Fos deficiency suppressed NV and reduced blood leakage during OIR. (**A**) Representative flat mount images depicted isolectin B4-stained retinal NV in *Nrl*^*Cre/+*^;*c-fos*^*f/f*^ and *c-fos*^*f/f*^ retinas at OIR P17. Inset images provided a closer view of neovascular tufts. Quantification images highlighted the areas of NV in red, and VO in yellow (*n* = 18–24). (**B**) Representative flat mount images displayed isolectin B4-stained retinal NV in *Rho*^*iCre/+*^;*c-fos*^*f/f*^ and *c-fos*^*f/f*^ OIR retinas at P17. Inset images offered a detailed view of neovascular tufts. Quantification images showed the areas of NV in red, and VO in yellow (*n* = 15–26). (**C**) Representative fundus fluorescein angiography (FFA) images exhibited retinal vasculature and leakage in the eyes of *Rho*^*iCre/+*^;*c-fos*^*f/f*^ and *c-fos*^*f/f*^ OIR and normal mice at P60 (*n* = 4). Data are represented as mean ± SEM. Mann-Whitney test was used for two-group comparison. ***, *p* < 0.001; **, *p* < 0.01; *, *p* < 0.05; ns, no significance. Scale bars in (A) and (B) are 1000 μm

To unravel the mechanism underlying how rod photoreceptor c-Fos regulates pathological development of NV in mice, we engineered two distinct rod photoreceptor specific *c-fos* deficient mouse lines (Fig. 2A). Given the opposing regulation of *c-fos* expression in the two phases of OIR (Fig. 1A), we employed the rhodopsin Cre (*Rho*^*iCre/+*^) mouse line [44] to delete c-Fos expression in late-stage rod development (*Rho*^*iCre/+*^;*c-fos*^*f/f*^). This aligns with the time point at which c-Fos is induced in OIR mice. Additionally, we utilized the neural retina leucine zipper protein (*Nrl*) Cre (*Nrl*^*Cre/+*^) mouse line [45] to delete c-Fos (*Nrl*^*Cre/+*^;*c-fos*^*f/f*^) in the rods and their precursors during the embryonic stage.

The *c-fos* floxed mice [46], previously validated [47], were crossbred with Cre mice. The *Rho*^*iCre/+*^ mice [44] represent a rod specific Cre line where a 4-kb mouse rod opsin promoter drives the expression of improved Cre (iCre) [48] recombinase in rods. Notably, iCre-mediated genomic DNA excision is detectable as early as at P7, with iCre protein expression reaching to 188 ± 44 ng/retina at P11 [44]. The *Nrl*^*Cre/+*^ mice [45] constitute another rod specific Cre line characterized by a 1.7 kb promoter upstream of *Nrl* exons 1 and 2, accompanied by a 724 bp intron, which governs Cre expression in rods and their precursors. Cre-mediated recombination can be detected from embryonic day 14 in rod precursors. The *Nrl*^*Cre/+*^ mice were employed to deactivate *c-fos* early in rod precursors and rods (*Nrl*^*Cre/+*^;*c-fos*^*f/f*^) to investigate the role of photoreceptor c-Fos in ROP development in the OIR model and normal vascular development (Fig. 2A). The *Rho*^*iCre/+*^ mice with rhodopsin promoter [44] were used to deactivate *c-fos* expression later in rods [44] (*Rho*^*iCre/+*^;*c-fos*^*f/f*^) to investigate the role of photoreceptor c-Fos in the retinal NV phase in the OIR model (Fig. 2B). The knockout efficiency of *c-fos* was validated at both mRNA and protein levels (Supplemental Fig. 1). It’s important to note that the photoreceptor specificities of Cre expression in *Nrl*^*Cre/+*^ and *Rho*^*iCre/+*^ mice had been previously confirmed [44, 45]. Our findings indicated that NV was significantly reduced in both *Nrl*^*Cre/+*^;*c-fos*^*f/f*^ and *Rho*^*iCre/+*^;*c-fos*^*f/f*^ OIR retinas in comparison to their respective control groups (Fig. 2A, B), highlighting the crucial role of rod c-Fos in the regulation of NV. Additionally, our observations revealed that rod c-Fos deficiency prior to phase I in *Nrl*^*Cre/+*^;*c-fos*^*f/f*^ OIR retinas led to a significant reduction in VO when compared to control retinas (Fig. 2A). We also noted a decrease in blood vessel leakage in *Rho*^*iCre/+*^;*c-fos*^*f/f*^ OIR mice lacking rod c-Fos (Fig. 2C). These results collectively suggest that the deletion of c-Fos expression in rod photoreceptor neurons inhibits the development of pathological NV and prevents retinal blood vessel leakage in OIR mice.

### c-Fos directly regulated Adam17 transcription

**Fig. 3:**
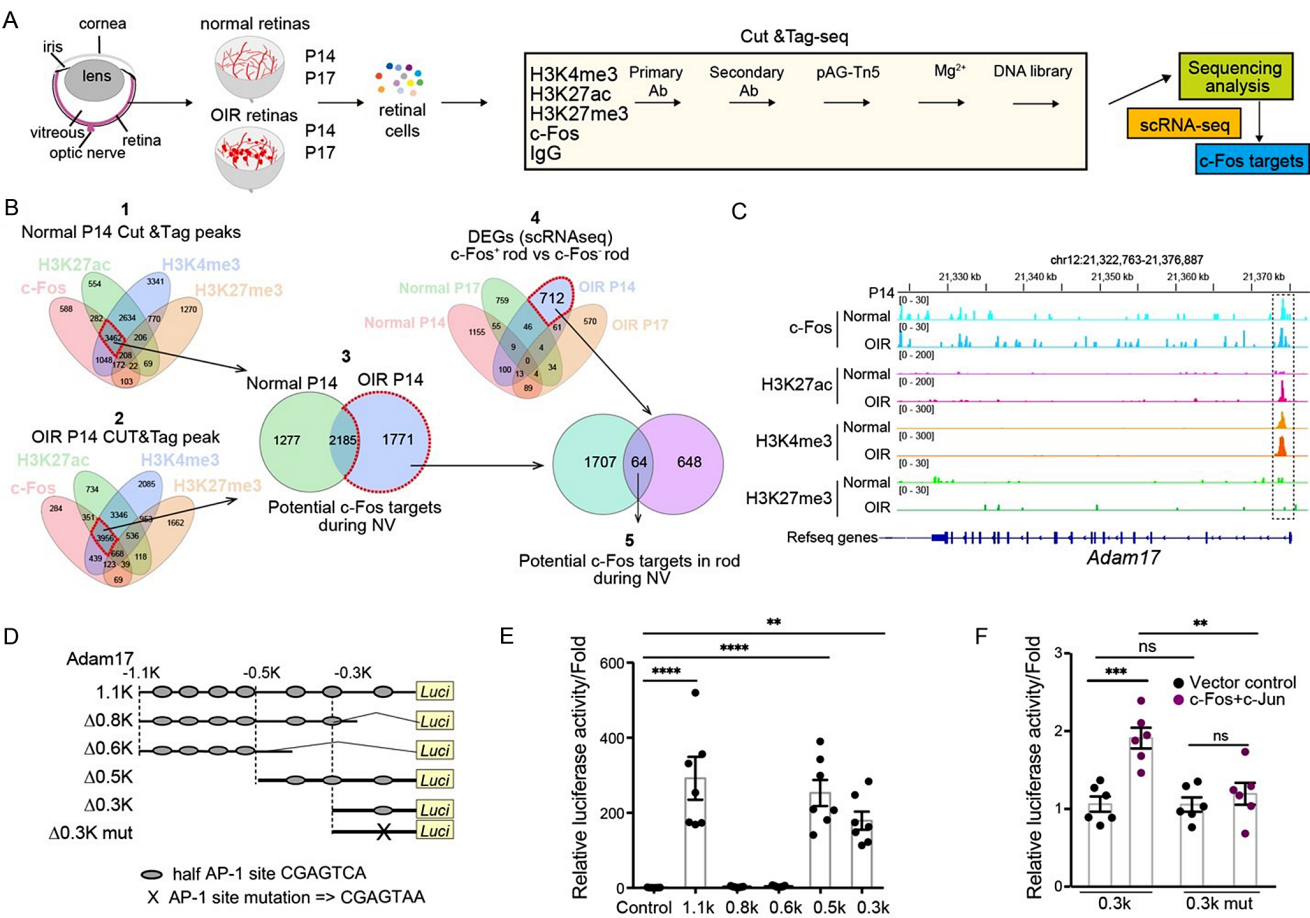
Adam17 was identified as a direct transcriptional target of c-Fos (**A**) Workflow for CUT&Tag sequencing of the retina from wild type OIR mice. (**B**) Venn diagrams: (1) Overlaps among genes with c-Fos peaks, H3K27ac peaks, H3K4me3 peaks, and H3K27me3 peaks within ± 1.0k bp from the transcription starting site (TSS) in P14 normal mice. (2) Overlaps among genes with c-Fos peaks, H3K27ac peaks, H3K4me3 peaks, and H3K27me3 peaks within ± 1.0k bp from the TSS in P14 OIR mice. (3) Overlaps of genes between c-Fos, H3K27ac, and H3K4me3, excluding H3K27me3 in both (1) and (2). (4) Overlaps of DEGs in c-Fos-expressing rod photoreceptors at P14 and P17, as well as during OIR at P14 and P17. (5) Intersection of peaks for only OIR P14 (3) and DEGs for only OIR P14 (4). (**C**) Representative genome browser tracks of c-Fos, H3K27ac, H3K4me3, and H3K27me3 CUT&Tag in the retinas of P14 normal and OIR mice for the *Adam17* gene. Red box indicated overlapped peaks; (**D**) Schematic of reporter plasmids. The 5’ region of the *Adam17* gene from TSS to -1.1k base pair was subcloned to the luciferase reporter plasmid. The AP-1 site deletion mutant reporters were created by restriction enzyme digestion. Gray circles indicate half AP-1 sites, and X denotes a mutated AP-1 site. (**E**) The reporter assay measures the promoter activity of the 5’ region of the *Adam17* gene from TSS to -1.1k bp, as well as that of deletion mutants (*n* = 6–7). (**F**) The reporter assay measures the promoter activity of the 5’ region of the *Adam17* gene from TSS to -300 bp, which contains an AP-1 binding site, with or without AP-1 (c-Fos and c-Jun), as well as with mutated AP-1 site (*n* = 6). Data are represented as mean ± SEM. Mann-Whitney test was used for two-group comparison. Kruskal-Wallis Dunn’s test was used for multiple-group comparison. ****, *p* < 0.0001; ***, *p* < 0.001; **, *p* < 0.01; ns, no significance

As a transcription factor, c-Fos forms a heterodimer known as the AP-1 complex when it interacts with c-Jun protein, thereby regulating the transcription of target genes. To elucidate which target genes of *c-fos* in rod photoreceptors contribute to NV development, we collected retinas from wild type normal and OIR retinas at P14 and P17. These retinas were then subjected to CUT&Tag sequencing (CUT&Tag-seq), utilizing a c-Fos antibody (with IgG as control) as well as antibodies for H3K27ac as a marker for active enhancers, H3K4me3 as a maker for active promoters, and H3k27me3 as a marker for gene repression (Fig. 3A). Given that most c-Fos CUT&Tag peaks were enriched near the transcription start sites (TSS) (Supplemental Fig. 2), we focused our analysis on the region ± 1.0 kb from the TSS. In this context, we identified 3,482 genes and 3,956 genes that exhibited overlapping c-Fos peaks, H3K24ac peaks, and H3K4me3 peaks, while excluding H3K27me3 peaks near the TSS in normal P14 and OIR P14 retinas, respectively (Fig. 3B). Out of these, 1,771 c-Fos potential target genes were expressed in OIR P14 (Fig. 3B). Next, we extracted the transcriptome data for rod photoreceptors from the OIR scRNA-seq dataset (GSE150703). We conducted an analysis of differentially expressed genes (DEGs), specifically comparing c-Fos positive and c-Fos negative rod photoreceptor cells. Our analysis identified 712 DEGs, particularly in OIR P14 (Fig. 3B). Interestingly, 64 of these 712 DEGs overlapped with the genes where c-Fos, H3K27ac, and H3K4me3 peaks coincided near the TSS in OIR P14 (Fig. 3B).

**Fig. 4:**
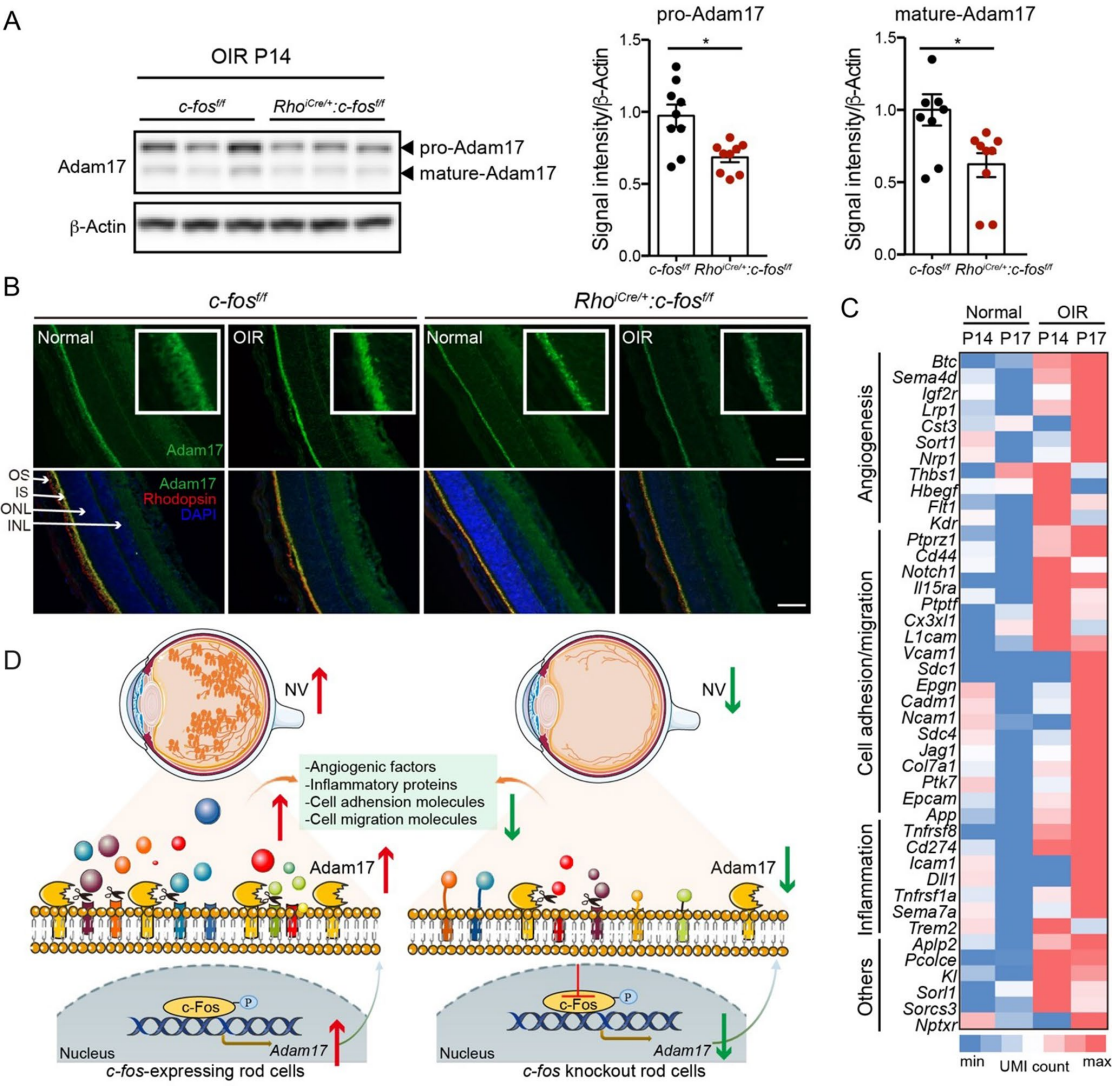
Photoreceptor c-Fos regulated pathological NV through Adam17-mediated pathways during OIR. (**A**) Western blots of pro-Adam17 and mature Adam17 in *Rho*^*iCre/+*^;*c-fos*^*f/f*^ mice and their littermate *c-fos*^*f/f*^ control mice. The levels of pro-Adam17 and mature Adam17 were determined by measuring the density of c-Fos bands and normalized them with β-Actin (*n* = 8–9). Data are represented as mean ± SEM. Mann-Whitney test was used for two-group comparison. *, *p* < 0.05. (**B**) Immunostaining of Adam17 on retinal cross-sections from *Rho*^*iCre/+*^;*c-fos*^*f/f*^ mice and their littermate *c-fos*^*f/f*^ control mice at P12 under normal condition and OIR retinas collected 4 h after exposure to room air from 75% oxygen at P12. Rhodopsin and DAPI were used for rod cells and cell nucleus staining, receptively. OS: Outer segment; IS: Inner segment; ONL: Outer nuclear layer; INL: Inner nuclear layer. (**C**) Heatmap displaying Adam17 substrates expressed in rod cells in normal and OIR mice at P14 and P17. (**D**) Model figure illustrating our discovery. c-Fos in the rod photoreceptors regulated Adam17 at the transcriptional level, leading to the production of bioactive inflammatory factors, angiogenic molecules, and cell adhesion and migration molecules that contribute to the pathological NV. Ablation of c-Fos can block the Adam17-mediated pathways, inhibiting abnormal blood vessel growth

**Fig. 5:**
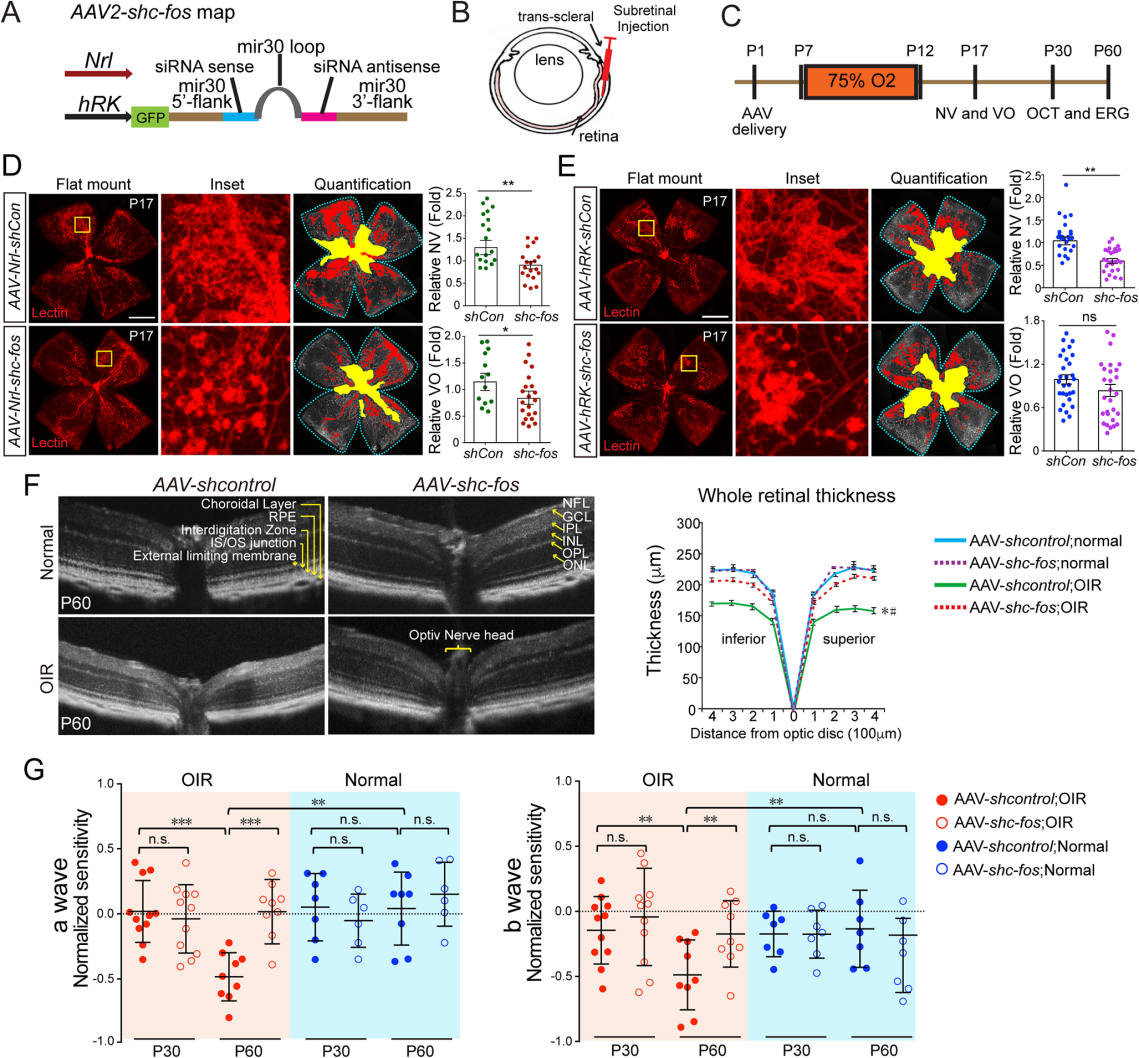
AAV-mediated rod c-Fos inhibition suppressed NV and improved retinal integrity and visual function. (**A**) Schematic representation of short hairpin *c-fos* (*shc-fos*) structures in adeno-associated virus (AAV); (**B**) Schematic illustration of subretinal injection procedure. (**C**) Workflow outlining the AAV injection analysis. (**D**) Representative flat mount images showing retinal NV in P17 OIR retinas with *AAV-Nrl-shcon* or *AAV-Nrl-shc-fos*. The inset images provide a higher magnification of neovascular tufts. The quantification images highlight the NV areas in red and VO areas in yellow (*n* = 14–18). (**E**) Representative flat mount images showing the NV in P17 OIR retinas with *AAV-hRK-shcon* or *AAV-hRK-shc-fos*. The inset images provide a higher magnification of neovascular tufts. The quantification images highlighted the NV areas in red and VO areas in yellow (*n* = 26–28). (**F**) Optical coherence tomography (OCT) scan of retinas with *AAV-hRK shcon* or *AAV-hRK shc-fos* in the eyes of P60 normal or OIR mice (*n* = 6). RPE: Retinal pigment epithelium; IS/OS: Junction of inner and outer photoreceptor segments; NFL: Nerve fiber layer; GCL: Ganglia cell layer; IPL: Inner plexiform layer; INL: Inner nuclear layer; OPL: Outer plexiform layer; ONL: Outer nuclear layer; Quantitative spider plot analysis illustrates the thickness of the retinas. (**G**) ERG analysis of the eyes from P30 and P60 normal or OIR mice with *AAV-hRK-shcon* or *AAV-hRK-shc-fos* (*n* = 9–11). Data are represented as mean ± SEM. Mann-Whitney test was used for two-group comparison. ***, *p* < 0.001; **, *p* < 0.01; *, *p* < 0.05; ns, no significance. Scale bars in (D) and (E) are 1000 μm

Among the 64 putative c-Fos targets in OIR P14 rod photoreceptor cells, a gene named a disintegrin and metalloprotease 17 (*Adam17*), also called tumor necrosis factor-alpha-converting enzyme (TACE) is a shedding protease responsible for inflammation and angiogenesis [49]. Our genome browser analysis of CUT&Tag tracks revealed a c-Fos peak that precisely overlapped with H3K27ac and H3K4me3 peaks at the TSS of the *Adam17* gene, while no such overlap with H3K27me3 peaks was observed at the TSS of *Adam17* gene (Fig. 3C). Intriguingly, the 5’ flanking region from TSS to -1.1 kb upstream of the *Adam17* gene contains a total of seven predicted AP-1 binding motifs (Fig. 3D), known to be essential sites for *c-fos/c-Jun* complex binding [50]. To dissect which of these AP-1 motifs were crucial for regulating the *Adam17* gene, we subcloned the 5’ flanking region from TSS to -1.1 kb, along with all seven motifs, into a luciferase reporter plasmid. Subsequently, we created deletion mutation, including ∆0.8 K, ∆0.6 K, ∆0.5 K, and ∆0.3 K, by progressively deleting from TSS to -0.3 kb, from TSS to -0.5 kb, from - 0.5 kb to -1.1 kb, and from - 0.3 kb to -1.1 kb, respectively (Fig. 3D). The reporter assay indicated that 1.1 K, ∆0.5 K, and ∆0.3 K exhibited similar promoter activity, whereas ∆0.8 K and ∆0.6 K lost promoter activity. This finding emphasized the significance of the region from TSS to -0.3k, which contains a single half AP-1 motif, for *Adam17* gene expression (Fig. 3E). Furthermore, a single nucleotide mutation of the AP-1 motif, changing TGAC to TTAC, within ∆0.3 K, abolished the enhancement of promoter activity by exogenous AP-1. This observation strongly suggests that c-Fos regulates *Adam17* gene expression through the proximal AP-1 motif near the TSS (Fig. 3F).

### Photoreceptor c-Fos regulated pathological NV through the Adam17-mediated pathways during OIR

In cellular context, Adam17 exists in two forms: an immature pro-form (pAdam17) and an active mature protease (mAdam17), responsible for its shedding activity [51]. Our in vitro findings suggest that *Adam17* is transcriptionally regulated by c-Fos. To investigate whether Adam17 expression changes occur in vivo, we examined retina tissues. Remarkably, we observed that the deletion of c-Fos in photoreceptor cells resulted in decreased levels of both pAdam17 and mAdam17 in the OIR retina at P14 (Fig. 4A). Additionally, we confirmed this decrease of Adam17 expression in photoreceptor layer in OIR retinas through immunostaining (Fig. 4B). These results strongly indicated that Adam17 is a direct target of c-Fos. In photoreceptor specific *c-fos* deficient mice, the reduction of Adam17 expression leads to an unbalanced pAdam17 to mAdam17 ratio that could further decrease shedding activity of mAdam17.

Adam17 is a membrane bound shedding metalloprotease with a crucial role in cleaving cell surface proteins, including growth factors, cytokines, receptors, and cell adhesion molecules. It plays a pivotal role in intracellular signaling pathways related to inflammation and angiogenesis [52–55]. By analyzing a publicly available scRNA-seq (Drop-seq) dataset (GSE150703) that compared OIR and normal conditions [56], we observed an increase in Adam17 substrates in rod photoreceptor cells in OIR mice (Fig. 4C). These substrates are involved in various aspects of pathophysiology of ROP including angiogenesis, inflammation, and cell adhesion as well as migration (Fig. 4C) [57, 58]. In summary, our data suggest that the upregulation of c-Fos expression in rod photoreceptors leads to transcriptional regulation of *Adam17*. This regulation results in the release of angiogenic and pro-inflammatory molecules through Adam17’s shedding activities, ultimately, promoting pathological NV in OIR (Fig. 4D).

Furthermore, in a previously study [5], we demonstrated that inflammatory factors, including TNFα, IL-6, and VEGFA, are regulated by c-Fos in *Vldlr*^*−/−*^ mice. To investigate whether these factors are also regulated by photoreceptor c-Fos in the OIR mouse model, we first examined the mRNA expression of these factors in whole retinas. Our findings revealed that rod c-Fos deficiency significantly reduced the induction of these factors during OIR compared to littermate control mice. Subsequently, we analyzed the peaks of c-Fos and histone markers using our CUT&Tag-seq data. However, no peaks of both c-Fos and active histone marker genes were identified within the promoter, enhancer, or gene body regions of these three factors (Supplemental Fig. 3). This suggests that these factors are indirectly regulated by c-Fos in the context of OIR.

### Inhibition of photoreceptor c-Fos via AAV suppressed retinal NV, preserved retinal integrity, and improved visual function

The use of RNA interference for therapeutic purposes has gained traction [59], and the recombinant AAV has been effectively employed in gene transfer therapies for various conditions, including congenital blindness [60–64]. In this study, we identified elevated c-Fos levels in rod photoreceptor cells as a contributing factor for pathological NV in OIR mice. To explore the translational potential of this discovery, we utilized adeno-associated virus (AAV) vector carrying shRNA targeting *c-fos* and employed a subretinal delivery approach to specifically suppress *c-fos* expression in rod photoreceptors and chose the shRNA sequences against *c-fos* whose efficacy have been previously validated [5]. Promoters including the *Nrl* promoter [65] and a well-documented human rhodopsin kinase (*hRK*) promoter in AAV therapy [66] were used to drive the suppression of *c-fos* expression in photoreceptors (Fig. 5A). Subretinal injections of AAV were administered on P1 (Fig. 5B), followed by the induction of OIR from P7 to P12. Retinal samples were collected on P17 for NV and VO analysis (Fig. 5C). Remarkably, both *AAV2-Nrl-shc-fos* and *AAV2-hRK-shc-fos* injections significantly suppressed the development of pathological NV when compared to the scramble control (Fig. 5D, E). Additionally, *AAV2-Nrl-shc-fos* treatment in mice led to decreased VO (Fig. 5D). Notably, it is important to mention that AAV-mediated c-Fos inhibition in rod photoreceptor did not influence normal retinal vascular development and knockdown efficacy and specificity were validated (Supplemental Fig. 4).

To further assess the viral therapeutic potential of viral-mediated photoreceptor c-Fos inhibition in retinal angiogenesis, we examined the recovery of retinal thickness and visual function in OIR mice following AAV treatment. Retinal thickness was assessed and analyzed using InSight software (Phoenix) as we described previously [14]. The thickness of the retinal layer had decreased in OIR retinas (Fig. 5F). Interestingly, *AAV2-hRK shc-fos* treatment preserved the attenuated retinal layer thickness at P60, without affecting retinal layer thickness of normal eyes (Fig. 5F). Photoreceptor visual function was assessed using scotopic ERG in both normal and OIR mice. There were no differences between *AAV2-hRK-shc-fos* and control groups in photoreceptor sensitivity (Fig. 5G) regarding a-wave and b-wave in normal mice at P30 and P60. However, when evaluating the protection effects of *AAV2-hRK-shc-fos* in OIR mice (Fig. 5G), we observed that both a-wave and b-wave sensitivities had declined in OIR mice at P60, but not at P30. Remarkably, *AAV2-hRK-shc-fos* treatment reinstated the attenuated a- and b-wave sensitivities to normal levels, suggesting the protection role of c-Fos inhibition in photoreceptor function. In summary, these findings suggest that targeting *c-fos* through AAV inhibition holds promise as a potential therapeutic strategy for ROP.

## Discussion

Proliferative blood vessel growth is the leading cause of blindness in vascular eye disease like ROP. It has been implicated that rod photoreceptors involved in ROP pathogenesis both in clinical research and animal models [16, 67, 68], but the underlying mechanisms remain elusive. In our current study, we have unveiled a novel mechanism involving the transcription factor c-Fos in rod photoreceptors, which plays a pivotal role in modulating pathological NV in the context of an OIR mouse model. First, we observed a significant upregulation and activation of c-Fos specifically in the photoreceptor layer during the initial stages of retina NV development. Second, we developed two distinct photoreceptor specific knockout mouse lines to investigate the precise role of photoreceptor c-Fos in controlling retinal NV. These models allowed us to delete c-Fos either before Phase I (utilizing Nrl-Cre) or before Phase II (employing Rho-iCre) of OIR progression. In both photoreceptor specific c-Fos knockout mice, the absence of c-Fos led to a significant reduction of retinal NV. This demonstrated the critical involvement of photoreceptor c-Fos in promoting pathological angiogenesis. In addition, deletion of photoreceptor c-Fos, especially before Phase I, also notably decreased VO, indicating the protection against vascular outgrowth by photoreceptor c-Fos early deficiency. Moreover, photoreceptor c-Fos deletion protected against blood vessel leakage compared to littermate control mice. Lastly, we identified Adam17 as a direct transcriptional target of c-Fos using CUT&Tag-seq and in vitro assays. Furthermore, we demonstrated a potential therapeutic approach for ROP by targeting photoreceptor c-Fos using AAV, emphasizing the clinical relevance of our finding. Together, our study established that photoreceptors play a crucial role in governing retinal NV through the c-Fos/Adam17 axis. These findings provided valuable insights into the intricate molecular mechanisms underlying pathological angiogenesis and hold promise for the development of innovative therapeutic strategies for ROP.

Retinal photoreceptor neurons have recently emerged as key players in the regulation of retinal NV in vascular eye diseases. For example, photoreceptors produce soluble inflammatory factors and induce inflammatory changes in nearby cells in diabetes [2–4] to mediate diabetes-induced degeneration of retinal vessels. Photoreceptor c-Fos controls retinal angiogenesis by modulating inflammatory signals in *Vldlr*^*−/−*^ mice [5], featuring neovascular aspect of human age-related macular degeneration [6–10]. However, whether photoreceptor neurons play any role in controlling ROP has not been reported yet. In this study, we demonstrated that photoreceptor neurons controlled retinal angiogenesis and inflammation via c-Fos by directly modulating the transcription of Adam17 that controls the cleavage of bioactive molecules in inflammation and angiogenesis such as cytokines and growth factors. This discovery has significant implications for our understanding of the pathophysiology of ROP as well as other vascular eye diseases and the development of novel therapeutic interventions.

There are two types of photoreceptor neurons: cones and rods. Cones develop early before birth; and rods are developed later than cones [69]. Multiple evidence in patients and animal studies have indicated the involvement of rod photoreceptor in the ROP pathology. It is known the age of onset of NV in ROP coincide with rod photoreceptor outer segment development [18, 70]. In animal models, it is reported that rod photoreceptors instigate the vascular abnormalities [67]. In clinical studies [71], ROP has fewer effects on the cone than on the rod responses, suggesting that cones are more resistant to the ROP disease process. The similar shape of the b-wave stimulus/response function in preterm infants and controls is evidence that ROP does not alter the balance of ON and OFF signals in the cone pathway [71]. Hence, ROP pathology likely originates from rod instead of cone photoreceptors. In this study, we used the rod photoreceptor specific promoter or Cre lines to generate rod-specific deficient mice to investigate the role of rod photoreceptors in controlling angiogenesis in ROP.

Systemic *c-fos*^*−/−*^ mice display normal ocular and retinal morphology when not exposed to acute light pulses [22], suggesting that c-Fos may not be required for retinal function under physiological condition. *c-fos* is induced by darkness in photoreceptors and the absence of *c-fos* prevents light-induced apoptotic cell death of photoreceptors in retinal degeneration [22]. In *Vldlr*^*−/−*^ mice, c-Fos is induced in photoreceptors and suppression of c-Fos inhibits retinal angiogenesis through suppression of inflammatory signals [5]. Our study showed that c-Fos is induced in the photoreceptor layer in the OIR mouse model and AAV targeted c-Fos inhibition with hRK promoter in rod photoreceptor didn’t influence the normal retinal vascular development.

Normally, the photoreceptor layer and the subretinal space are in an immune privilege zone in which adaptive immunity and inflammation are highly controlled [72, 73], maintained by immunosuppressive factors and characterized by a lack of immune cells and therefore, tolerance of foreign antigens [73, 74]. Eye diseases with pathological angiogenesis have a slow “para-inflammatory” response [75, 76] with changes in adaptive immune cells and macrophage infiltration violating the normal ocular environment. Loss of immune privilege may correlate with and increase NV [77, 78]. Inflammatory cytokines, including IL6, IL1β, TNFα, and TGFβ are produced by a variety of cell types, particularly macrophages and monocytes. Recent studies show that inflammatory signals from photoreceptors control retinal angiogenesis in diabetic retinopathy [2–4] and *Vldlr*^*−/−*^model [5]. In this study, we assessed whether these inflammatory signals originate from photoreceptors to control NV in ROP mouse model via c-Fos. c-Fos is a transcription factor containing a consensus DNA element, TGA (C/G) TCA, defined as the 12-*O*-Tetradecanoylphorbol-13-acetate-Responsive Element (TRE) [50, 79]. TREs serve as the binding platform for Fos and Jun family members [80]. Our published data [5] suggest that multiple inflammatory factors (TNFα, IL6, and VEGFA) are regulated in c-Fos-enriched retinas. We analyzed the proximal promoter sequences of these genes and found that all of them contain one or more potential TREs. In this study, we also observed the regulation of these inflammation factors by photoreceptor c-Fos. Therefore, we examined whether c-Fos can bind TREs on the promoters of TNFα, IL6, and VEGFA to directly control their expression in OIR retinas using our CUT&Tag sequencing dataset, but none of them are actively binding with c-Fos during NV, suggesting that photoreceptor c-Fos may regulate these factors indirectly.

Adam17 is a ubiquitously expressed protein in retina from P7, including ganglion cell layer, inner and outer plexiform layers, and photoreceptors [81], it is reported that its shedding activity is required for the retinal angiogenesis [82]. In the retinas of diabetic retinopathy patients and mouse model, the expression and enzymatic activity of Adam17 are upregulated, with its chemokines substrates such as CXCL16 and CX3CL1 [83, 84], and inactivation of endothelial Adam17 or its genetic inhibitor, the tissue inhibitor of matrix metalloproteinases-3 (TIMP3) ameliorates pathogenesis both in diabetic retinopathy and retinal ischemia reperfusion [83, 85–87], indicating an important role of Adam17 in vascular ocular pathologies. As a shedding enzyme, Adam17 have a broad spectrum of substrates involve in cytokines production, cell-cell communication and signaling receptors cleavage, play important roles in inflammation, angiogenesis, cell adhesion and migration [88–90]. We utilized a published scRNA-seq dataset to demonstrate the increased presence of Adam17 substrates in photoreceptors of OIR mice. These substrates were found to predominantly govern inflammation, angiogenesis, and cell adhesion/migration, which are known to be the contributing factors to the ROP pathology. Nevertheless, it remains to be determined whether these substrates also play a role in retinal angiogenesis independently of the shedding activity of Adam17. Furthermore, we identified Adam17 as a direct transcriptional target of c-Fos in retinal photoreceptor neurons and both pro- and active- form of Adam17 were reduced in photoreceptor specific c-Fos deficient retinas during OIR. These findings suggest that photoreceptors may control pathological retinal angiogenesis potentially through a novel pathway involving c-Fos/Adam17 pathway. However, the direct targeting of Adam17 as a contributor to c-Fos-mediated pathological retinal angiogenesis remains inconclusive. Additional experiments are required to explore the interaction between c-Fos and Adam17. For instance, investigating whether the absence of Adam17 can mitigate c-Fos-induced pathological retinal angiogenesis and studying the impacts of Adam17 inhibition on the activation/shedding of molecules such as *Tnf, Il6, Vegfa*, and other inflammatory factors during pathological NV in an OIR mouse model. How those substrates of Adam17 are regulated by c-Fos/Adam17 axis in photoreceptors during OIR also need to be further studied.

In this study, we have uncovered significant insights into the role of transcription factors, particularly their upregulation in rod photoreceptors in OIR mice. Through targeted ablation of c-Fos specifically in rods, we successfully inhibited the pathological angiogenesis observed in the OIR retina. Our investigation also delved into the intricate mechanistic details, revealing that c-Fos acts as a pivotal regulator of Adam17, an enzyme responsible for cleaving bioactive molecules associated with inflammation, angiogenesis, and cell adhesion and migration. This regulatory pathway was found to contribute significantly to the development of pathological NV in OIR mice. Expanding upon our discoveries, we conducted translational research, demonstrating that the selective c-Fos inhibition by AAV rod photoreceptors could effectively suppress pathological NV and, importantly, enhance visual outcomes in mice. These findings not only advance our understanding of retinal vascular diseases but also offer a paradigm shift by emphasizing the active role of photoreceptor neurons in controlling retinal NV in vascular eye diseases. This newfound perspective underscores the intricate interplay between neuronal and vascular components within the retina and opens exciting possibilities for the development of innovative, precisely targeted treatments. Ultimately, this research holds immense potential to revolutionize the management of retinal vascular diseases, ultimately benefiting countless individuals at risk of vision loss.

### Electronic supplementary material

Below is the link to the electronic supplementary material.

Supplementary Material 1

## Data Availability

All the data supporting the conclusions of this study are included within the article and supplementary data. All the other data and materials are available upon request to the corresponding author. The CUT&Tag-seq dataset is deposited to the Genome Expression Omnibus (GEO) under the accession numbers (GSE245456).
