# Supplementary material for: Photoreceptors inhibit pathological retinal angiogenesis through transcriptional regulation of Adam17 via c-Fos
Source: Angiogenesis. 2024 Mar 14;27(3):379–95. doi: 10.1007/s10456-024-09912-0 (PMC11303108; doi:10.1007/s10456-024-09912-0)
Supplement: Supplementary file 1 — Supplementary Material 1 [file 10456_2024_9912_MOESM1_ESM.docx]

**Supplementary Figures and Tables**


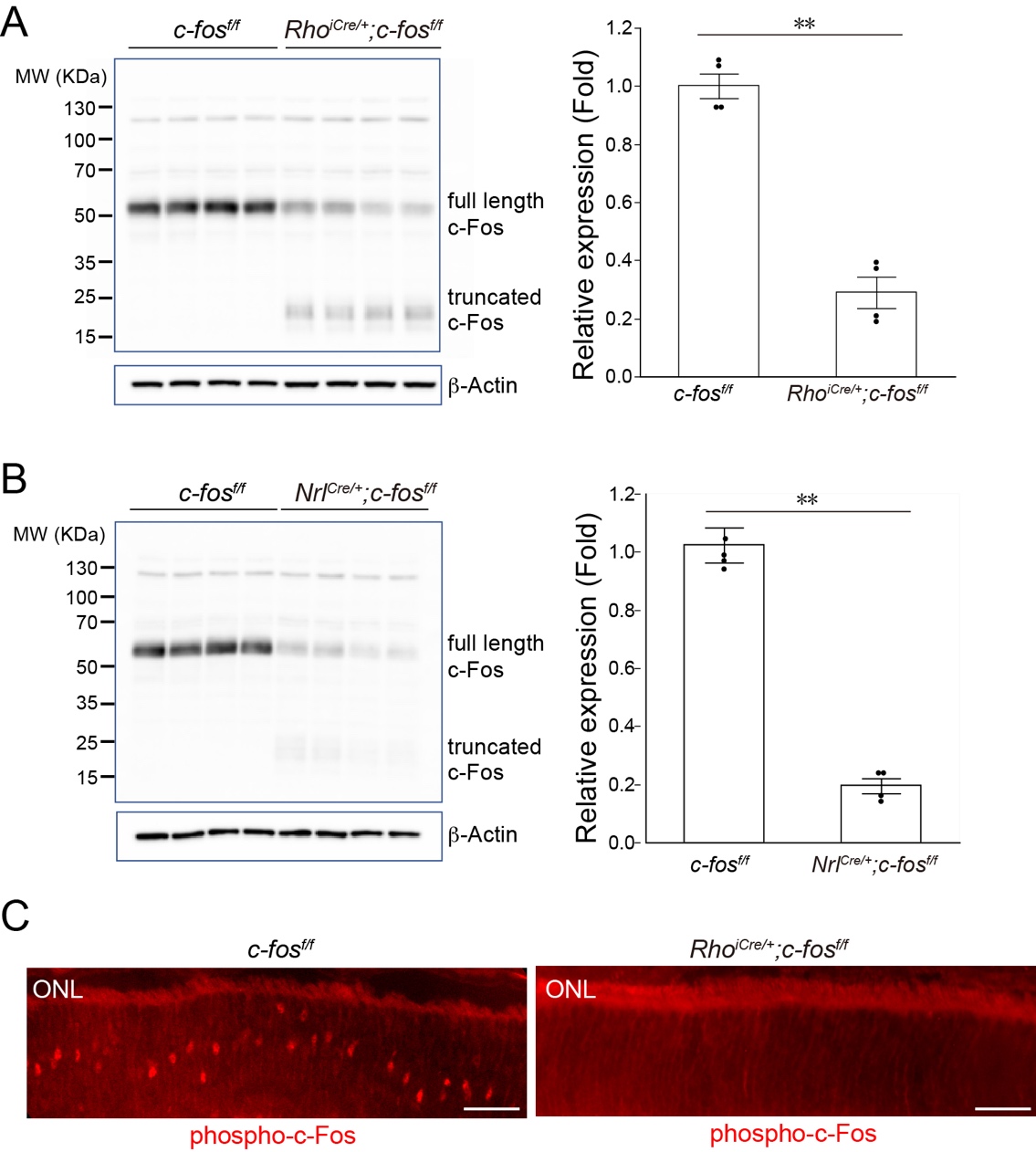


**Figure S1.** Rod photoreceptor-specific c-Fos knockout mice were validated. (A) Western blots were conducted to examine c-Fos protein expression in retinas from *Rho^iCre/+^;c-fos^f/f^* mice and littermate *c-fos^f/f^*  control mice. The levels of c-Fos were determined by quantifying the density of c-Fos bands and normalized them to β-Actin (n=4). (B) Western blots were conducted to examine c-Fos protein expression in retinas from from *Nrl^Cre/+^;c-fos^f/f^* mice and littermate *c-fos^f/f^*  control mice. The levels of c-Fos were determined by quantifying the density of c-Fos bands and normalized them to β-Actin(n=4). (C) Immunofluorescence staining was employed to visualize c-Fos phosphorylation (phosphor-c-Fos, red) in the ONL of OIR retinas from *c-fos^f/f^*  and *Rho^iCre/+^;c-fos^f/f^* mice. Data are represented as mean ± SEM. Mann-Whitney test was used for two-group comparison*. **, p<0.01*.


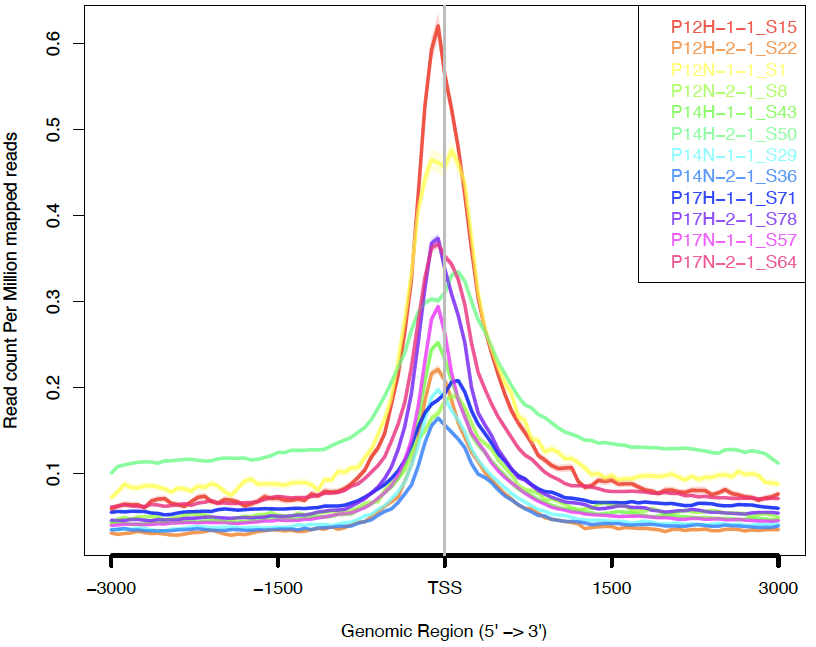


**Figure S2.** Enrichments at TSS of the CUT&Tag libraries. Read distributions across peaks. The x-axis represents regions near peaks; the y-axis represents the normalized read counts. 0: peak center; -3000: 3 kb upstream of the peak center, 3000: 3 kb downstream of the peak center.


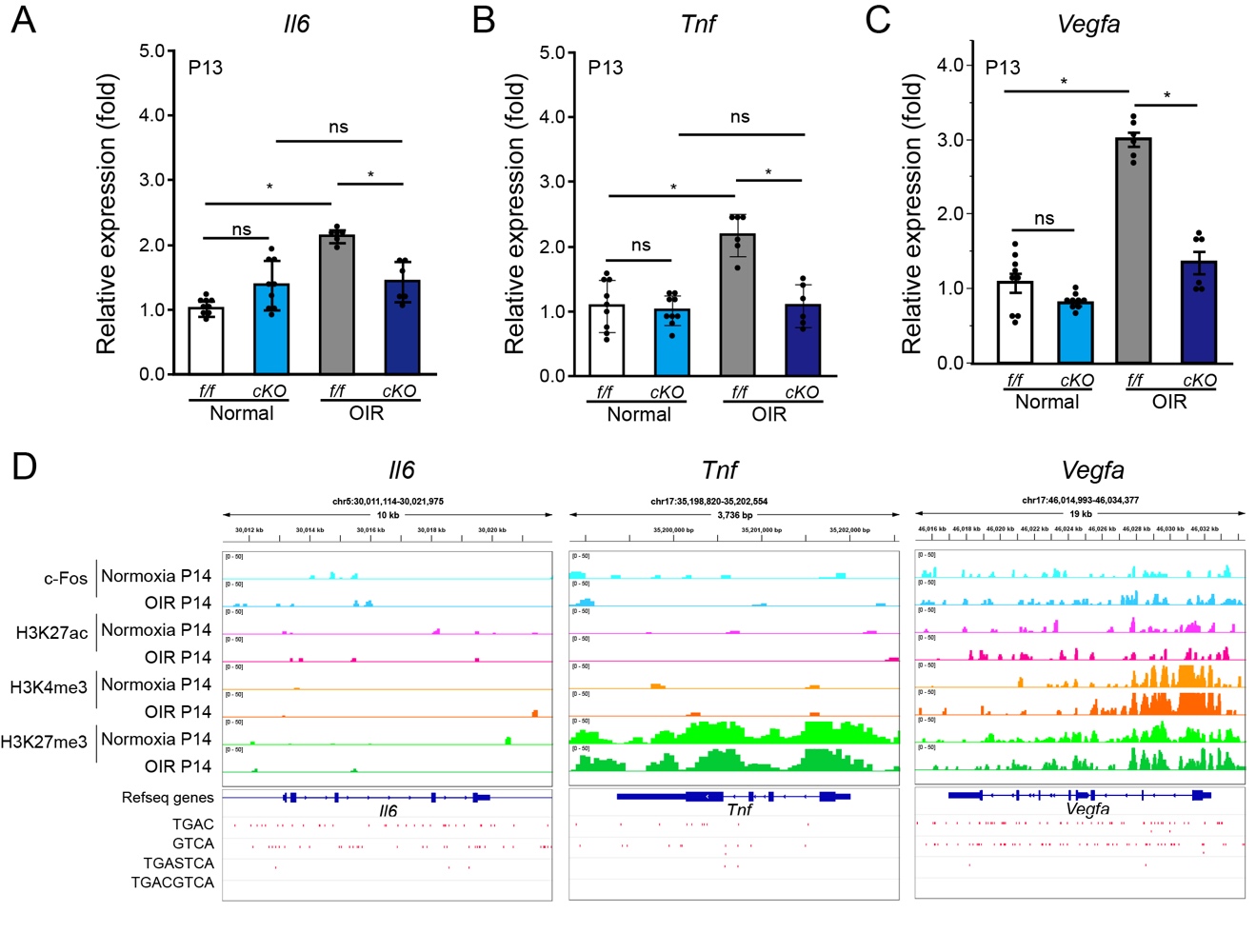


**Figure S3.** The mRNA expression of inflammatory factors in normal and OIR mice. (A-C) The mRNA levels of inflammatory factor including *Il6, Tnf,* and *Vegfa* were assessed in whole retinas from both *Rho^iCre/+^;c-fos^f/f^* mice and *c-fos^f/f^* mice under normal and OIR conditions at P13 using RT-qPCR (n=6-8). (D) Representative genome browser tracks of c-Fos, H3K27ac, H3K4me3, and H3K27me3 CUT&Tag in the retinas of P14 normal and OIR mice for *Il6, Tnf,* and *Vegfa* genes. Data are represented as mean ± SEM. Mann-Whitney test was used for two-group comparison. Kruskal-Wallis Dunn’s test was used for multiple-group comparison. **, p<0.05;* ns, no significance.


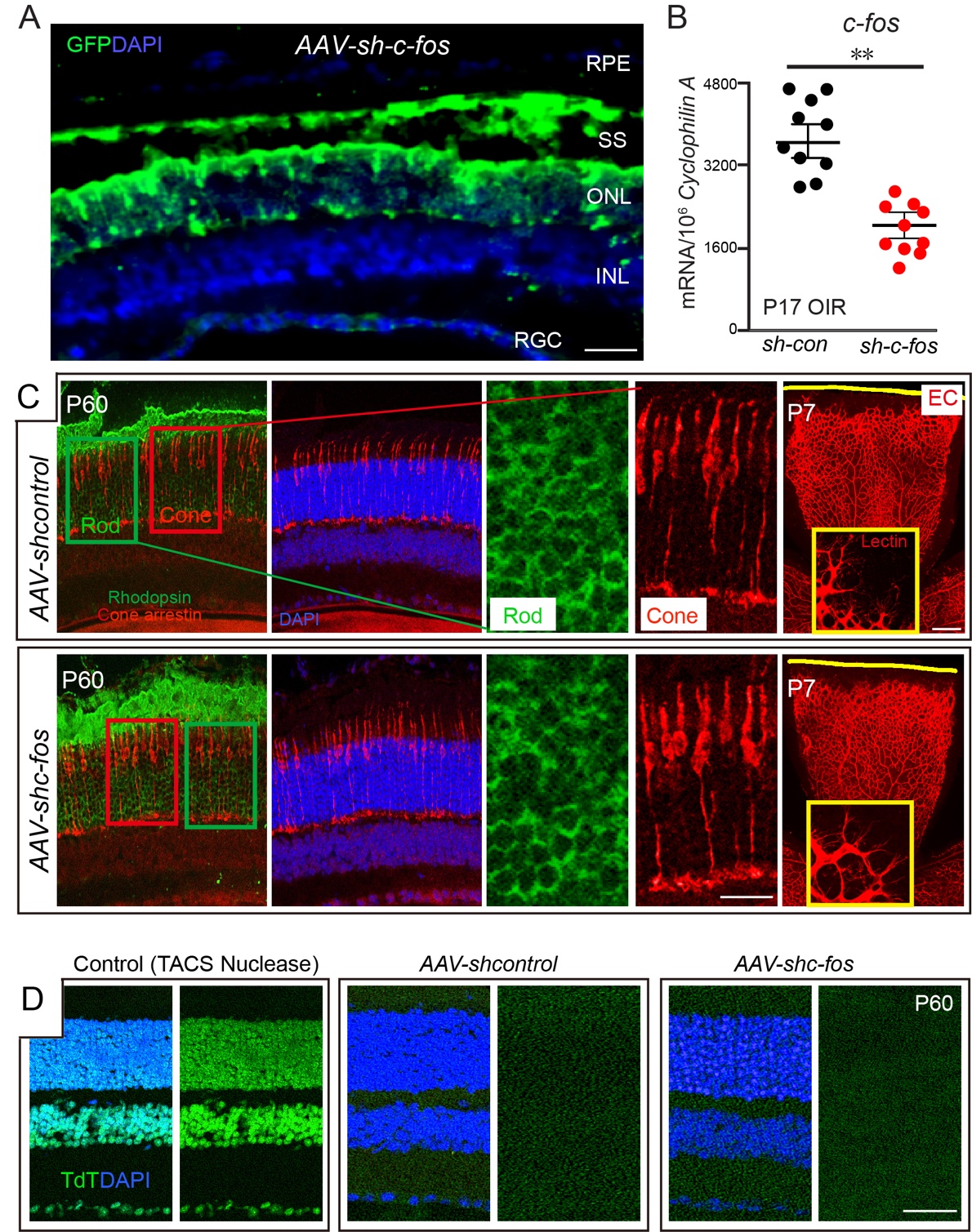


**Figure S4.** shRNA targeting c-Fos had no discernible impact retinal development. (A) Immunofluorescence staining on retinal cross sections showed the photoreceptor specificity of *AAV-hRK-sh-c-fos*; (B) The mRNA expression of *c-fos*  showed the knockdown efficacy of *AAV-hRK sh-c-Fos* in retinas (n=10); (C) Immunofluorescence staining of rod photoreceptor cells, cone photoreceptor cells, and endothelial cells (EC) with anti-Rhodopsin, anti-Cone arrestin, and isolectin B4, respectively, in the retinas of mice infected with *AAV-hRK-shc-fos* or *AAV-hRK-shcontrol*. (D) TUNEL assay of retinas infected with *AAV-hRK shc-fos* or *AAV-hRK-shcontrol* at P60. TUNEL, green. Data are represented as mean ± SEM. Mann-Whitney test was used for two-group comparison. ***, p<0.01;* ns, no significance.

**Table S1.** Antibodies for IHC and CUT&Tag (company, clone number, cat #, RRID#)

| **Antibodies** | **Vender** | **Catalog number** | **RRID number** | **Usage** |
| --- | --- | --- | --- | --- |
| Histone H3K4me3 | Active Motif | 39159 | AB_2615077 | CUT&Tag |
| Histone H3K27ac | Active Motif | 39133 | AB_2561016 | CUT&Tag |
| guinea pig anti-rabbit IgG | Antibodies-Online | ABIN101961 | AB_10775589 | CUT&Tag |
| Tri-Methyl-Histone H3 (Lys27) (C36B11) | Cell Signaling Technology | 9733S | AB_2616029 | CUT&Tag |
| c-Fos (9F6) | Cell Signaling Technology | 2250S | AB_2247211 | CUT&Tag, WB, IHC |
| Anti-Adam17 | Abcam | ab13535 | AB_300436 | WB, IHC |
| β-Actin (D6A8) | Cell Signaling Technology | 8457S | AB_10950489 | WB |
| Phospho-c-Fos (Ser32) (D82C12) | Cell signaling | 5348S | AB_10557109 | IHC |
| Rhodopsin, clone 4D2 | Millipore | MABN15 | AB_10807045 | IHC |
| Isolectin GS-IB4 Alexa Fluor™ 594 Conjugated | Fisher | I21413 |  | IIHC |
